# Toll like Receptors (TLRs) expression in HIV children

**DOI:** 10.1186/1471-2334-14-S3-O10

**Published:** 2014-05-27

**Authors:** M Madeshwaran, V Gupta, J Chakrabarty, G Narayan, A Kumar

**Affiliations:** 1 Department of Pediatrics, IMS, BHU, Varanasi, India; 2 Department of Medicine, IMS, BHU, Varanasi, India; 3 Department of Molecular and Human Genetics, Faculty of Science, BHU, Varanasi, India

## Background

Immune activation plays vital role in HIV infection and further progression to AIDS, with TLRs primarily involved in this mechanism.

## Methods

Twenty eight children with HIV in age group of 18 months-14 years attending anti retroviral clinic and 17 healthy children as controls were included in the study. WHO clinical staging, CD4+ count and Toll like receptors (TLRs) 1, 7, 8, 9 expressions from PBMC (peripheral blood mononuclear cells) were studied and correlated with various parameters.

## Results

Mean age of study population was 8.08 ± 3.41 years. 75% of patients were in WHO clinical stage I and II, whereas 25% in stage III. 29% HIV children had severe immunosuppression. TLR-1 showed neutral expression in majority (96%) of HIV children. For TLR-7, 53.5% HIV children showed upregulation, whereas 32% and 14.5% showed downregulation and neutral expression, respectively. TLR-8 expression was downregulated in majority (93%) of HIV children. TLR-9 was upregulated in 29% of patients, whereas 71% had neutral expression. Of all the TLRs studied, only TLR-9 had correlation with WHO clinical stage 3 and severe immunosuppression. No correlation of TLR expression was found with age, nutritional status and ART status of patients.

## Conclusion

Expression pattern of TLR-7 was similar to adult patients with HIV. ART had no effect on expression profile of TLRs. Children with severe immunosuppression had significant up regulation of TLR-9. Similar correlation was not observed with TLR-1, 7 and 8. Altered expression pattern of TLR might have implications for disease progression in HIV infection.

